# The phosphoinositide regulatory network in *Trypanosoma brucei*: Implications for cell-wide regulation in eukaryotes

**DOI:** 10.1371/journal.pntd.0008689

**Published:** 2020-10-29

**Authors:** Igor Cestari, Kenneth Stuart

**Affiliations:** 1 Institute of Parasitology, McGill University, Sainte-Anne-de-Bellevue, Quebec, Canada; 2 Division of Experimental Medicine, McGill University, Montreal, Quebec, Canada; 3 Center for Global Infectious Disease Research, Seattle Children’s Research Institute, Seattle, Washington, United States of America; 4 Department of Global Health, University of Washington, Seattle, Washington, United States of America; Hunter College, CUNY, UNITED STATES

## Abstract

The unicellular eukaryote *Trypanosoma brucei* undergoes extensive cellular and developmental changes during its life cycle. These include regulation of mammalian stage surface antigen variation and surface composition changes between life stages; switching between glycolysis and oxidative phosphorylation; differential mRNA editing; and changes in posttranscriptional gene expression, protein trafficking, organellar function, and cell morphology. These diverse events are coordinated and controlled throughout parasite development, maintained in homeostasis at each life stage, and are essential for parasite survival in both the host and insect vector. Described herein are the enzymes and metabolites of the phosphatidylinositol (PI) cellular regulatory network, its integration with other cellular regulatory systems that collectively control and coordinate these numerous cellular processes, including cell development and differentiation and the many associated complex processes in multiple subcellular compartments. We conclude that this regulation is the product of the organization of these enzymes within the cellular architecture, their activities, metabolite fluxes, and responses to environmental changes via signal transduction and other processes. We describe a paradigm for how these enzymes and metabolites could function to control and coordinate multiple cellular functions. The significance of the PI system’s regulatory functions in single-celled eukaryotes to metazoans and their potential as chemotherapeutic targets are indicated.

## Introduction

**Fig 1:**
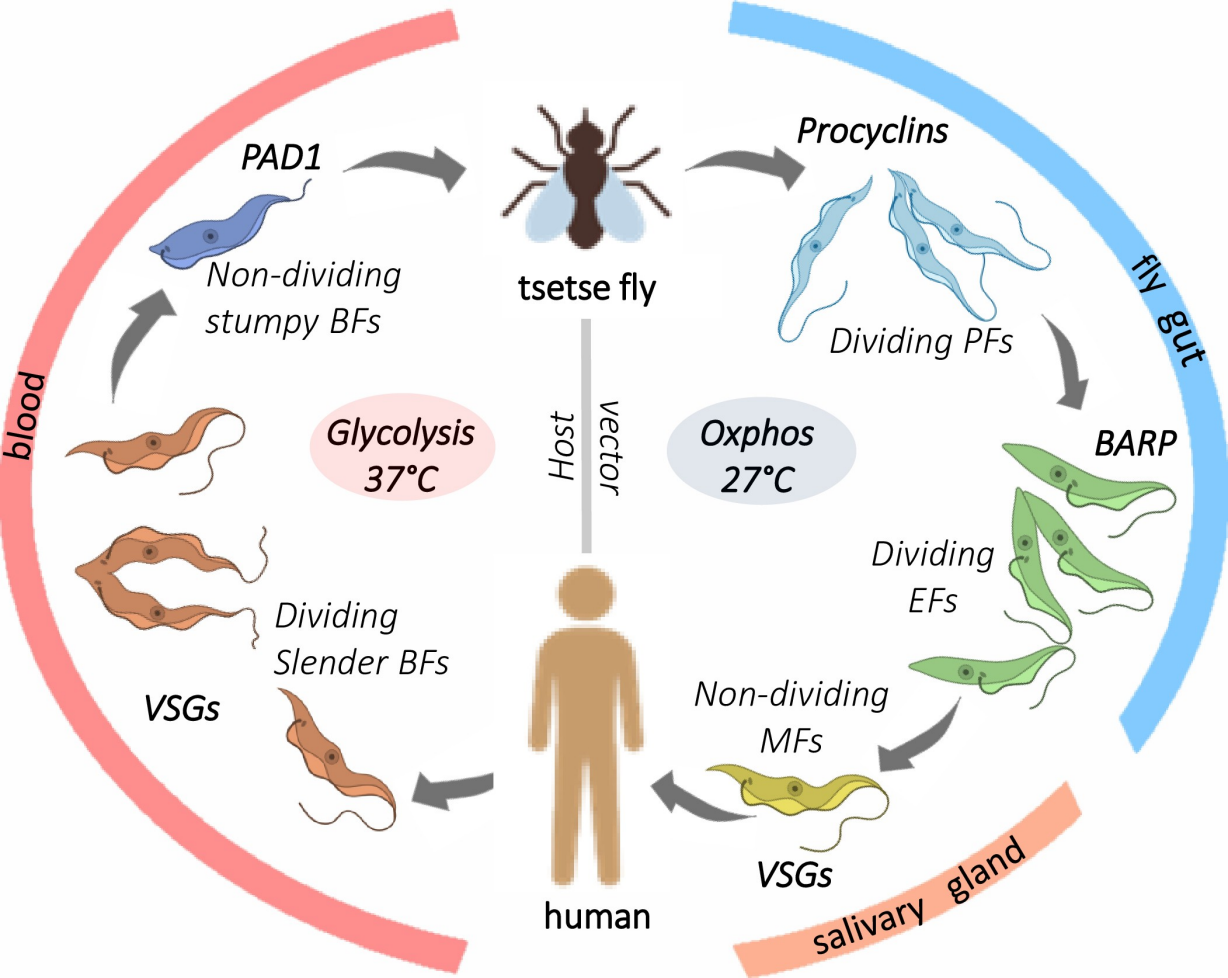
*brucei* life cycle indicating characteristic gene expression differences between the stages. **Diagram of *T*.** The slender BFs proliferate in the blood, express VSGs, and metabolize glucose as the main energy source and develop into nondividing stumpy BFs that cease VSG expression and express PAD1. The stumpy forms develop into PFs that proliferate in the fly gut, express EPs and GPEETs, and primarily generate energy via oxphos. The PFs develop into EFs in the salivary gland that express BARPs and subsequently develop into infective MFs that resume VSG expression. BARPs, brucei alanine-rich proteins; BFs, bloodstream forms; EFs, epimastigote forms; EPs, procyclin rich in Glu-Pro repeats; GPEETs, procyclin rich in Glu-Pro-Glu-Glu-Thr repeats; MFs, metacyclic forms; oxphos, oxidative phosphorylation; PAD1, proteins associated with differentiation 1; PFs, procyclic forms; VSGs, variant surface glycoproteins.

**Fig 2:**
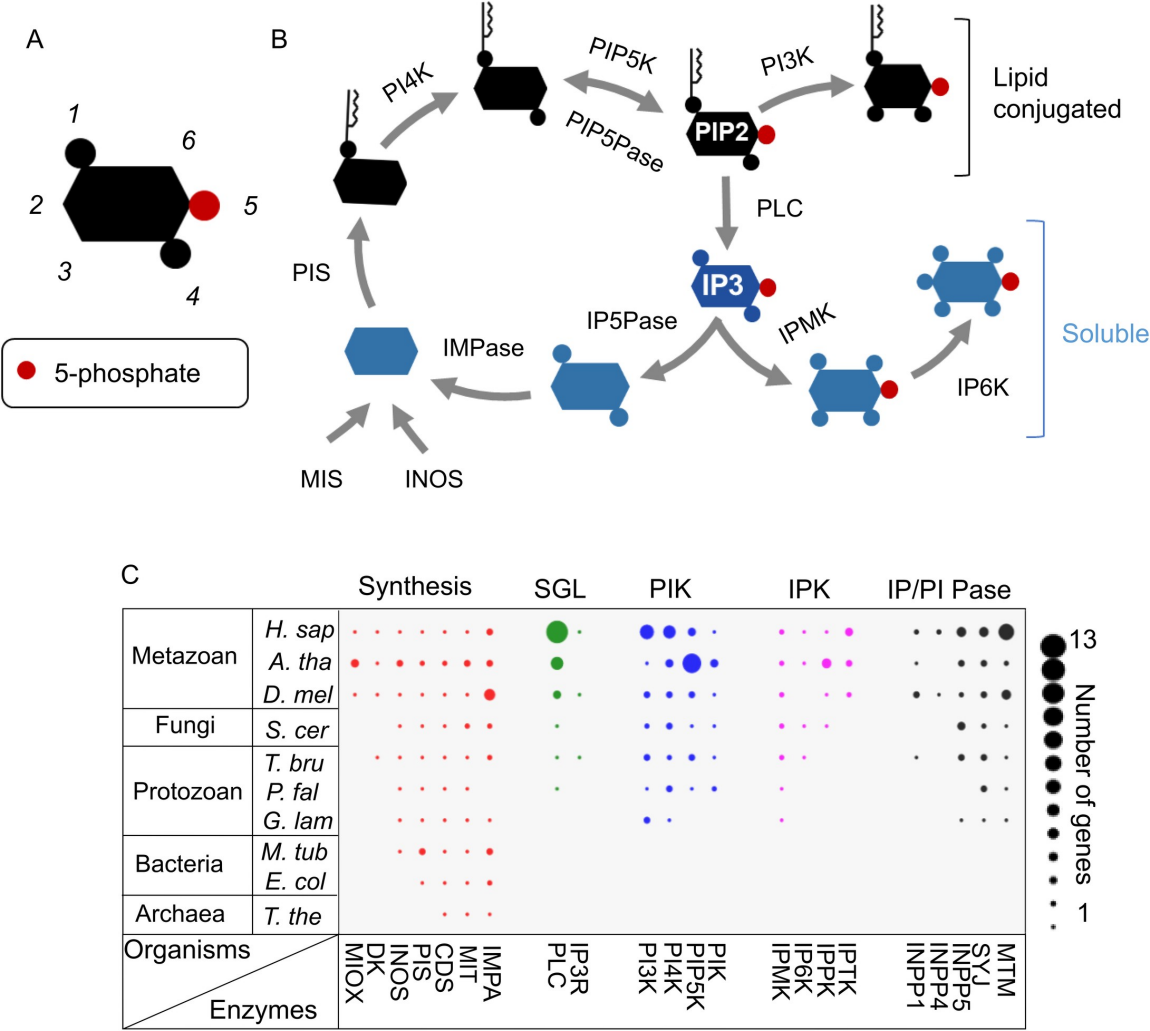
Overview of the PI system in *T*. *brucei*. (A) Diagram showing numbering of inositol positions that can be phosphorylated, dephosphorylated, or pyrophosphorylated. Positions 1, 4, and 5 are phosphorylated in IP3 (position 5 is shown in red) and is shown as an example. (B) Simplified diagram showing lipid conjugated (in black) and soluble (in blue) metabolites and enzyme activities for the metabolite interconversions. See Table 1 for the list of the enzymes (and their abbreviations) found in *T*. *brucei*. PLC cleavage releases IP3 from the lipid. Inositol can be synthesized from glucose-6-phosphate by INOS or transported as MIS, and PI is synthesized by PIS. (C) PI gene homologs in prokaryotes, protozoa, fungi, and metazoa. The numbers of homologous PI genes are indicated by the circle sizes in each organism. The general roles of the enzymes in synthesis, SGL, PI, or IP kinase (PIK or IPK) and IP or PI phosphatase (IP/PI Pase) are labeled and represented by different colors. IP/PI Pase indicate enzymes that can dephosphorylate IPs and/or PIs. The organisms are *Homo sapiens (H*. *sap)*, *Arabidopsis thaliana (A*. *tha)*, *Drosophila melanogaster (D*. *mel)*, *Saccharomyces cerevisiae (S*. *cer)*, *T*. *brucei (T*. *bru)*, *Plasmodium falciparum (P*. *fal)*, *Giardia lamblia (G*. *lam)*, *Mycobacterium tuberculosis (M*. *tub)*, *Escherichia coli (E*. *col)*, and *Thermus thermophilus (T*. *the)*. CDS, CDP-diacylglycerol synthetase; DK, diacylglycerol kinase; IMPA, Inositol-1-monophosphatase; IMPase, inositol (1,4) monophosphatase; INOS, inositol-3-phosphate synthase; INPP1, inositol polyphosphate-1-phosphatase; INPP4, inositol polyphosphate-4-phosphatase; INPP5, inositol polyphosphate-5-phosphatase; IP, inositol phosphate; IPK, inositol polyphosphate kinase; IPMK, inositol polyphosphate multikinase; IPPK, inositol-pentakisphosphate 2-kinase; IPTK, inositol trisphosphate 5/6 kinase; IP3, inositol trisphosphate; IP3R, inositol 1,4,5-triphosphate receptor; IP5Pase, inositol polyphosphate 5-phosphatase; IP6K, inositol hexakisphosphate kinase; MIOX, myo-inositol oxygenase; MIS, myo-inositol-1-phosphate synthase; MIT, myo-inositol/proton symporter; MTM, phosphatidylinositol-3-phosphatase myotubularin; PI, phosphatidylinositol; PIK, phosphatidylinositol kinases; PIP2, phosphatidylinositol 4,5-bisphosphate; PIP5K, phosphatidylinositol 4-phosphate 5-kinase; PIP5Pase, phosphatidylinositol 5-phosphatase; PIS, phosphatidylinositol synthase; PI3K, phosphatidylinositol 3-kinase; PI4K, phosphatidylinositol 4-kinase; PLC, phospholipase C; SGL, signaling; SYJ, phosphatidylinositol 5-phosphatase synaptojanin.

All cellular functions are governed by complex integrated regulatory systems that control and coordinate numerous processes. The unicellular eukaryote *Trypanosoma brucei* undergoes extensive developmental changes during its life cycle (Fig 1). *T*. *brucei* cells maintain cellular homeostasis in each proliferative state and periodically differentiate unidirectionally via nondividing developmental intermediates into the next proliferative stage. They proliferate as slender bloodstream forms (BFs) in the animal host, change into nondividing stumpy BF developmental intermediates, which differentiate into proliferative procyclic forms (PFs) in the midgut of the tsetse fly insect vector. The PFs develop, via less well-characterized stages, into epimastigote forms (EFs) that colonize the fly salivary glands and produce animal infective metacyclic forms (MFs). The developmental processes that occur during the parasite life cycle entail changes in surface composition, gene expression, metabolism, organelle composition and function, and cell morphology (Fig 1). These developmental changes result in differentiated states that are adapted for survival in the host and vector rather than being reversible physiological shifts in response to environmental changes. Multicellular eukaryotes, e.g., humans, have many differentiated cell types and thus have a more complex regulatory system than single-celled organisms such as *T*. *brucei* [1]; nevertheless, the cellular regulatory systems in both *T*. *brucei* and multicellular eukaryotes control cellular homeostasis in each differentiated state. The regulation enables essential activities such as growth and division and responses to environmental changes at each life stage while also providing for developmental changes and differentiation into other stages. Analysis of the developmental changes in *T*. *brucei*, which are simpler than in multicellular eukaryotes, albeit still complex, provides an opportunity to develop insights into cell-wide regulatory processes that may be evolutionarily conserved. The phosphatidylinositol (PI) and inositol phosphate (IP) enzymes and their small molecule substrates and products (Fig 2A and 2B and Table 1) constitute a complex cellular regulatory network (called the PI system hereafter for simplicity) that controls numerous cellular processes. It does so in conjunction with the other cellular regulatory networks and regulates essential cellular processes such as transcription, mRNA turnover, organelle biogenesis, energy metabolism, and cell division and development. It also integrates these cellular processes with responses to environmental changes (e.g., in the host and vector) and thus is part of a complex system of regulatory networks. The PI system enzymes and metabolites are highly organized within the cell and are not only catalysts, substrates, and products but also importantly are regulatory effectors. They function in part by binding to proteins and changing their conformation, activity, and/or interactions, thereby regulating cellular functions. The PI system enzyme homologs are conserved between *T*. *brucei* and multicellular eukaryotes. Still, they are expanded in number and diversified in the latter (Fig 2C), where they perform regulatory functions as well as catalytic interconversions that are specific to each differentiated cell type. The processes that occur within the single *T*. *brucei* cell during its developmental transitions provide an attractive system to elucidate fundamental features of complex regulatory networks in eukaryotes and how they control and coordinate many cellular processes in an integrated fashion.

**Table 1:** PI system genes of *T*. *brucei*.

Function	Abbreviation	Product name	Gene ID	Location	Essentiality	References
**Synthesis**	MIT	Myo-inositol/proton symporter	Tb927.11.5350	PM/Gg	Yes (BF/PF)	[99,100]
	INOS	Inositol-3-phosphate synthase	Tb927.10.7110	Cy^π^	Yes (BF)	[101]
	CDS	CDP-diacylglycerol synthetase	Tb927.7.220	PM/ER/Gg*	Yes (BF)	[2,90]
	DK	Diacylglycerol kinase	Tb927.8.5140	-	-	-
	PIS	CDP-diacylglycerol inositol 3-phosphatidyltransferase	Tb927.9.1610	ER/Gg	Yes (BF)	[35]
**SGL**	PLC	Phosphoinositide-specific phospholipase C	Tb927.11.5970	PM/Fl	No (BF)	[2,36]
	IP3R	Inositol 1,4,5-trisphosphate receptor	Tb927.8.2770	Ac	Yes (BF)	[37]
**Kinases**	PIP5K 1	Phosphatidylinositol 4-phosphate 5-kinase	Tb927.4.1620	PM	Yes (BF/PF)	[2,7]
	PIP5K 2	Phosphatidylinositol-4-phosphate 5-kinase	Tb927.10.4770	Fl*	No (BF)	[34]
	PIP5K 3	Phosphatidylinositol-4-phosphate 5-kinase	Tb927.10.3890	-	-	-
	PIP5K 4	Phosphatidylinositol 4-phosphate 5-kinase, putative	Tb927.7.6910	-	-	-
	PIP5K 5	Phosphatidylinositol 4-phosphate 5-kinase related	Tb927.1.740	Mt/kDNA^π^	-	-
	PI3P5K	Phosphatidylinositol 3-phosphate 5-kinase	Tb927.11.1460	En/Ls	Yes (BF)	[5]
	PI3K 1	Phosphatidylinositol 3-kinase catalytic subunit	Tb927.11.15330	Cy/En^π^	-	-
	PI3K 2	Phosphatidylinositol 3-kinase	Tb927.8.6210	Gg	Yes (BF)	[6]
	PI4K 1	Phosphatidylinositol 4-kinase alpha	Tb927.3.4020	Cy/FP^π^	-	-
	PI4K 2	Phosphatidylinositol 4-kinase beta	Tb927.4.1140	Gg	Yes (PF)	[93]
	IPMK	Inositol polyphosphate multikinase	Tb927.9.12470	-	Yes (BF)	[2,3,34]
	IP5K	Inositol pentakisphosphate kinase	Tb927.4.1050	Cy^π^	-	-
	IP6K	Inositol hexakisphosphate kinase	Tb927.7.4400	-	-	*-*
**Phosphatases**	PIP5Pase	Phosphatidylinositol (4,5 or 3,4,5)-phosphate 5-phosphatase	Tb927.11.6270	Nu	Yes (BF)	[2,4]
	IP1Pase	Inositol polyphosphate 1-phosphatase	Tb927.8.7170	Fl/FP*	No (BF)	[2–4]
	IMPase 1	Inositol-1(or -4)-monophosphatase 1	Tb927.9.6350	-	No (BF)	[2–4,34]
	IMPase 2	Inositol-1(or -4)-monophosphatase 2	Tb927.5.2690	Cy/Fl^π^	No (BF)	[2–4,34]
	IP5Pase 1	Inositol polyphosphate 5-phosphatase 1	Tb927.10.5510	Cy^π^	No (BF)	[3,34]
	IP5Pase 2	Inositol polyphosphate 5-phosphatase 2	Tb927.9.5680	Cy/En^π^	No (BF)	[34]
	IP6Pase	Inositol hexakisphosphate phosphatase	Tb927.8.3410	En^π^	-	-
	SYJ 1	Synaptojanin 1 (IP/PI 5-phosphatase)	Tb927.9.10640	Cy/En^π^	No (BF)	[34]
	SYJ 2	Synaptojanin 2 (IP/PI 5-phosphatase)	Tb927.7.3490	Cy	No (BF)	[3,34]
	SYJ 3	Synaptojanin 3 (IP/PI 5-phosphatase)	Tb927.11.5490	-	Yes (BF)	[3,5,34]
**PIK related**	TOR1	Phosphatidylinositol 3-kinase-related target or rapamycin (TOR) 1 protein	Tb927.10.8420	Nu	Yes (BF)	[102]
	TOR2	Phosphatidylinositol 3- or 4-kinase-related TOR 2 protein	Tb927.4.420	Mt/ER	Yes (BF)	[102]
	TOR3	Phosphatidylinositol 4-kinase-related TOR 3 protein	Tb927.4.800	Cy	Yes (BF/PF)	[103]
	TOR4	Phosphatidylinositol 3-kinase-related TOR 4 protein	Tb927.1.1930	Cy/En^π^	Yes (BF)	[104]
	TOR-like 1	FAT domain/Rapamycin binding domain/Phosphatidylinositol 3- and 4-kinase, putative	Tb11.v5.0793	-	-	-
	TOR-like 2	Phosphatidylinositol 3-kinase tor, putative	Tb11.v5.0744	-	-	-
	ATM	Phosphatidylinositol (3,4) kinase-related ataxia telangiectesia-mutated protein	Tb927.2.2260	Cy^π^	-	*-*
	ATR	Phosphatidylinositol 3-related kinase ATM-related	Tb927.11.14680	Nu^π^	-	-

**Fig 3:**
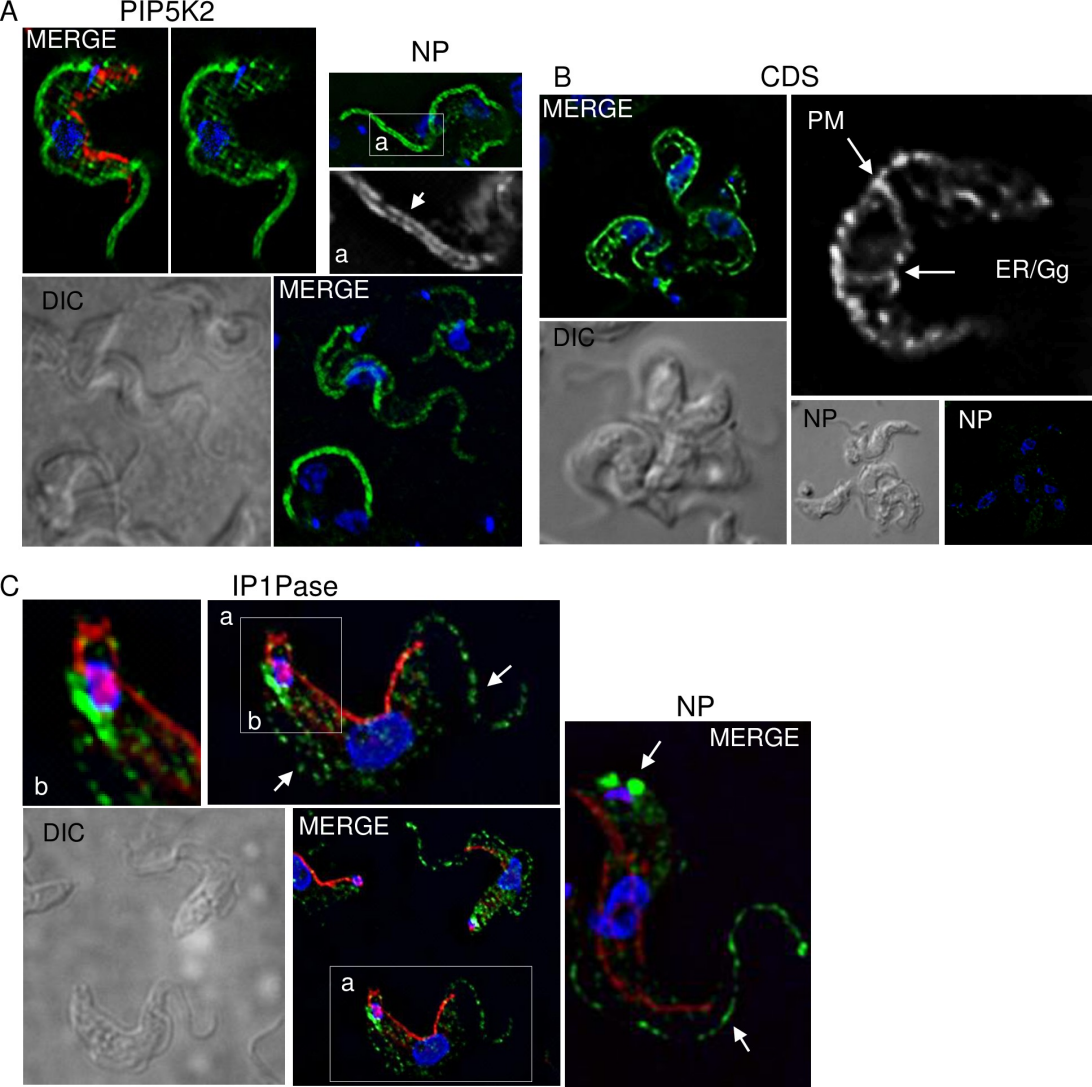
Subcellular immunofluorescence localization of PI enzymes in *T*. *brucei* slender BF. V5-tagged PIP5K2 (A), CDS (B), or IP1Pase (C) were expressed from rDNA locus upon induction with tetracycline and stained green with anti-V5 fluorescein-conjugated monoclonal antibodies. Mitochondria are stained red with mitoTracker, DNA is stained blue with DAPI, and DIC indicates differential interference contrast. Arrows indicate PI enzyme staining in black and white in expanded regions. The cells were permeabilized with 0.2% NP40 except as indicated as NP. BF, bloodstream form; CDS, CDP-diacylglycerol synthetase; DAPI, 4′,6-diamidino-2-phenylindole; DIC, differential interference contrast; ER, endoplasmic reticulum; Gg, Golgi; IP1Pase, inositol polyphosphate 1-phosphatase; NP, non-permeabilized; PI, phosphatidylinositol; PIP5K2, phosphatidylinositol 4-phosphate 5-kinase 2; PM, plasma membrane.

The general functions of the enzymes are indicated (SGL = signaling), and some are also diagrammed in Fig 3. The subcellular locations in BF and/or PF cells where known are ER, endoplasmic reticulum; Gg, Golgi; PM, plasma membrane; Fl, flagellum; Ac, acidocalcisome; Cy, cytoplasm; Nu, nucleus; FP, flagellar pocket, Mt, mitochondrial; kDNA, kinetoplast DNA; En, endocytic; Ls, lysosomes. Essentiality as determined by gene expression knockdown where known is also indicated.

*For this work, see Fig 1.

^π^Localization in PFs according to Tryptag (http://tryptag.org).

BF, bloodstream form; PF, procyclic form; PI, phosphatidylinositol; PIK, phosphatidylinositol kinase.

## Control and coordination of cellular processes

The PI system in *T*. *brucei* controls multiple cellular processes that maintain the detailed characteristics of each life cycle stage [2–7]. In the mammalian host, the BFs grow, divide, and proliferate in an environment that has a relatively constant temperature and biochemical composition except for clinical and immune responses. The proliferating slender BFs are abundant in the blood but also are present in the skin and adipose tissue and invade the central nervous system [8,9]. BFs generate energy by glycolysis, which occurs in specialized peroxisomes, the glycosomes [10,11]. They have an incomplete oxidative phosphorylation (oxphos) system and maintain mitochondrial membrane potential by adenosine triphosphate (ATP) hydrolysis [12]. Some oxphos components are encoded in mitochondrial DNA and undergo RNA editing, a complex guide RNA directed process that recodes the mRNAs [13]. Differential mitochondrial mRNA editing between life cycle stages results in different compositions of the oxphos system in these stages [14–18]. The metabolic changes, morphological, and numerous gene expression differences between BFs and PFs indicate that the regulatory systems control many cellular processes. Prominent among these is the control of cell surface composition. The BF surface is covered with a dense variant surface glycoprotein (VSG) coat, which is periodically changed by VSG gene recombination or by switching transcription to a different VSG [reviewed by [19]]. This results in antigenic variation and evasion of the host immune response. The *T*. *brucei* genome encodes approximately 2,500 VSG genes, but only 1 is expressed at a time and from telomeric expression sites (ESs). The ESs contain several co-transcribed ES-associated genes (ESAGs), except those ESs that are expressed in MFs. Unusually, ESs are transcribed by RNA polymerase I (RNAP I) and transcription initiates from a promoter located 40 to 60 kb upstream of the VSG gene [reviewed by [19]], which is developmentally regulated [4,20,21]. Thus, as detailed below, the surface composition and its variation, mitochondrial, and cytoplasmic energy generating systems and the expression of multiple genes are controlled by the PI system in concert with other cellular regulatory networks.

Morphologically distinct nondividing stumpy BFs occur periodically in vivo and in vitro in a strain-dependent fashion. Stumpy BFs development is initiated via quorum-sensing mechanisms that involve oligopeptides binding to a surface G-protein coupled receptor family of protein [22,23]. Unlike slender BFs, the stumpy BFs express carboxylate transporter proteins associated with differentiation 1 (PAD1) and PAD2 [24,25], nicotinamide adenine dinucleotide hydrogen (NADH) dehydrogenases (diaphorase) [25,26], edit mitochondrial mRNAs for some components of the oxphos system, which is nevertheless incomplete in this stage [15,27,28], and express other metabolic components [17,29]. They are thus primed to develop into PFs that occurs upon transmission of BFs to the tsetse fly. PFs do not express VSGs but, instead, express abundant procyclin proteins on their surface. The PFs are larger than BFs, have an expanded mitochondrion with a complete oxphos system, metabolize glucose outside the glycosomes, and have numerous gene expression and mRNA editing differences from BFs [3,27,30] [reviewed by [31]]. For example, mitochondrial cytochrome b and cytochrome oxidase subunit 2 mRNAs are edited and functional in PFs but are not edited in slender BFs [15,16,18]. Reciprocally, only a small 5′ regions of NADH subunit 7 (ND7) mRNAs is edited in PFs, but ND7 mRNAs are fully edited in BFs [14,32]. The vast differences between BFs and PFs, along with the fact that PFs do not survive at 37°C, indicate that the transition from BFs to PFs constitutes differentiation rather than a physiological shift. The PFs proliferate in the tsetse fly midgut then, as has been described elsewhere [33], develop into other stages at various locations in the fly and ultimately develop in the fly salivary glands into mammalian infective MFs (Fig 1). As summarized below, the PI system functions in the control of multiple cellular processes in *T*. *brucei* that maintain the homeostatic, albeit responsive, state in each life cycle stage as well as the development from one stage to another. Importantly, the PI system is integrated within the network of other cellular regulatory processes, e.g., protein kinases and phosphatases, which regulate multiple cellular processes in *T*. *brucei* as well as in other eukaryotes.

## The *T*. *brucei* PI system

A total of 30 PI system genes were identified in *T*. *brucei* (and in the related *Trypanosoma cruzi* and *Leishmania* parasites) by BLAST analysis of PI genes; conserved domains; and catalytic amino acids from Archaea, prokaryotes, protozoans, yeast, worms, insects, plants, and humans (summarized in Fig 2C) [2]. The annotations of genes encoding PI enzymes in the kinetoplastid genome database (TritrypDB.org) were confirmed but also identified other genes annotated as hypothetical proteins [34]. *T*. *brucei* thus encodes 13 kinases, 10 phosphatases, 1 phospholipase C (PLC), 1 D-myo-inositol 1,4,5-triphosphate (IP3) receptor (IP3R), and it also encodes enzymes involved in the synthesis and recycling of PI metabolites, including a myo-inositol transporter (Table 1). An additional 8 PI kinase–related enzymes have been identified [e.g., target of rapamycin (TOR)-related enzymes], but it is unknown whether the PI3 or PI4 kinase domain in these *T*. *brucei* enzymes is functional. Confirmation of the catalytic specificities predicted by homology is incomplete. The predicted functions of some PI enzymes have been confirmed by enzymological analysis, e.g., inositol polyphosphate multikinase (IPMK), which phosphorylates IP3 and D-myo-inositol 1,3,4,5-tetrakisphosphate (IP4) at inositol positions 3 and 6, respectively [3,34] and by a combination of gene knockdowns and biochemical analysis such as mass spectrometry or metabolic labeling, e.g., CDP-diacylglycerol inositol 3-phosphatidyltransferase [35] and phosphatidylinositol 4-phosphate 5-kinase (PIP5K) [2,7]. A notable exception is phosphatidylinositol (4,5 or 3,4,5)-phosphate 5-phosphatase (PIP5Pase), which was annotated as an IP3 5-phosphatase. Enzymological analysis using purified *T*. *brucei* protein combined with active site mutations that disrupt catalysis showed that this protein dephosphorylates the 5-phosphate position of phosphatidylinositol 4,5-bisphosphate (PIP2) and phosphatidylinositol 3,4,5-triphosphate (PIP3) but not of IP3 [2,4].

The subcellular locations, abundances, and activities of the PI enzymes have been partially determined (Table 1). The current data suggest organizational features consistent with their function in a regulatory network. The PI system enzymes are located in various cellular compartments, e.g., plasma membrane and flagellum [2,36], flagellar pocket [7], Golgi [6], acidocalcisomes [37], and nucleus [2,4]; and in some cases have more than one location [2,7]. This implies that the substrate and product interconversions of the respective metabolites occur at these subcellular locations. Moreover, transcriptomics and ribosomal profiling studies indicate that the abundances of some enzymes differ between *T*. *brucei* life cycle stages [30]. The activities of the PI system enzymes are likely to be subject to various regulatory controls, such as feedback regulation mediated by the substrates and products, or by processes such as protein phosphorylation. This organization is also likely to be affected by the interactions of the PI proteins with the other cellular components and by dynamic cellular processes.

How might such a regulatory network control homeostasis and development? A likely answer resides in the known ability of PI metabolites to specifically bind to proteins and affect protein functions and interactions with other proteins [3,4,38–44]. The binding of these metabolites by cellular proteins affects protein catalytic activities and interactions or functions within multiprotein complexes [4,43–46]. Importantly, the various PI metabolites have unique combinations of phosphorylated and pyrophosphorylated positions on the inositol moiety due to the PI enzyme catalytic activities (see Fig 2B). This provides for their specific molecular interactions with target proteins. Such proteins thus contain binding sites that are specific for certain PI metabolites, e.g., the Pleckstrin homology (PH) domains, with a distinct affinity of interaction [38,47,48]. In addition, the phosphoinositides have a diacylglycerol and a lipid constituent with hydrophobic properties that enable interaction with membranes or other hydrophobic domains, whereas the IP is soluble in aqueous environments. The extant knowledge suggests that the PI system is conserved among unicellular and multicellular eukaryotes [41,42,47,49–53]; it controls multiple processes in trypanosomes [2,3,6,7,34,37], and it has related functions in other eukaryote pathogens [54–56].

Affinity purification and mass spectrometry analysis have identified hundreds of proteins that bind to IP3, IP4, and PIP3 in *T*. *brucei* [3], and numerous proteins bind to PIs in mammalian cells and cells of other organisms [39,57]. Many studies in various cell systems have shown that the proteins that bind PIs have various functions including those involved in gene expression regulation [4,45,58]; signaling pathways [50,55,59,60]; cell metabolism [3,61]; vesicle trafficking; and protein synthesis, folding, and degradation [3,5–7]. The location of the PI enzymes and metabolites in different cellular compartments suggests that they function as a cell-wide integrated system as opposed to a biochemical pathway that is restricted to metabolic interconversions. While some functions of the PI system are conserved among trypanosomes, yeast, and metazoans, such as the control of protein trafficking [5,6,46–48,62], others evolved to be specific to trypanosomes such as the control of antigenic variation [2,4]. The differences in PI regulatory functions reflect divergence in the regulatory systems among eukaryotes. Trypanosomes notably diverged early in the eukaryote lineage and have specialized regulatory processes, such as mitochondrial RNA editing. They also rely heavily on posttranscriptional regulatory mechanisms to control gene expression, which reflects their lack of specific transcription initiation factors, unusual polygenic transcription, and the almost exclusive use of *trans*-splicing for mRNA processing [63]. This implies that studies of the PI system in *T*. *brucei* may identify important and novel aspects of eukaryotic posttranscriptional regulation. The amounts, locations, and interactions of the PI enzymes in trypanosomes are highly organized within this parasite’s architecture, and their activities are subject to various types of regulation that affect and are likely influenced by the levels and fluxes of their substrates and products. Such an arrangement thus provides a dynamic cellular regulatory network that embodies numerous specific molecular interactions and functions.

## A paradigm

A paradigm for the role of the PI system in the coordinated regulation of multiple cellular processes in *T*. *brucei* and other eukaryotes is based on 3 types of results. **(1) The PI system is highly organized.** The PI system is highly ordered within the cell with the locations and amounts of PI enzymes determined by a combination of their primary sequences and the cellular processes that control their translation, subcellular location, and turnover. We surmise that the activities of the enzymes are controlled by other processes such as those that affect protein folding, modification (e.g., phosphorylation), local levels of their substrates and products, and interactions with other cellular constituents such as proteins and membranes. This dynamic system thus controls local subcellular fluxes of specific metabolites in a manner that is responsive to internal and external conditions. **(2) Proteins bind to specific PI metabolites.** Various proteins specifically bind PI metabolites, and some but not all PI interacting domains have been characterized. The specificity of the interaction is due to the phosphorylation state of the metabolite, access to the target, and the target’s competence to bind the metabolite, which may be affected by additional molecular interactions. Additionally, metabolite flux and abundance, as well as the affinity of the interaction, can affect the protein and PI interaction. **(3) PI metabolites regulate protein function.** The consequence of protein and PI binding is that the activity, binding to other molecules (e.g., other proteins), is affected, and this has downstream effects, e.g., a cascade of molecular interactions. The binding of PIs to multiple specific protein targets affects target functions and provides for coordination of the functional consequences, e.g., coordination of diverse processes. The specific coordination would be enhanced/modulated by the physical segregation of the enzymes and metabolite transporters. Hence, this highly organized system can coordinately regulate multiple processes at the level of functional activity analogous to the kinase/phosphatase regulation of protein activity and/or interactions to which control the expression of numerous genes via transcription factors in response to signal transduction.

The PI regulatory system may be metastable, i.e., react in a substantially predetermined fashion in response to certain conditions. For example, host conditions that arise in response to parasite infection and/or proliferation (e.g., quorum sensing) may alter the PI regulatory network resulting in the development of slender to stumpy BFs. The location of PI enzymes on the plasma membrane, e.g., PIP5K and PLC, indicates that they may function in the sensing and reacting to the relevant condition. The BF might have alternative fates: remaining in the host and undergoing an antigenic switch if not eliminated by the immune response or developing into a PF if ingested by the tsetse fly. Both alternatives have clear selective value since they enhance parasite survival. Also, such a system would enable stumpy BFs to be advantageous but not obligate developmental intermediates. In any event, a metastable regulatory system would keep the parasites poised to switch antigenic type or develop into the PF stage. This regulatory system would also provide for control of the several developmental stages in the insect and host.

## Key processes regulated by the PI system in *T*. *brucei*

The role of the PI system in controlling multiple cellular processes is evident from cellular changes following the genetic perturbation of PI enzymes in *T*. *brucei* [2,3,5,6,37]. Among these processes are the regulation of VSG expression and switching, life stage development, energy metabolism, protein traffic, and organelle maintenance and function.

## Nuclear phosphoinositides and the control of VSG expression

In *T*. *brucei*, phosphoinositides play an essential role in the control of VSG gene allelic exclusion and antigenic switching [2,4]. *T*. *brucei* expresses only one of the hundreds of VSG genes at a time and periodically changes its expression by switching transcription between VSG ESs or by VSG recombination mechanisms. Multiple molecules are implicated in this VSG regulation and switching, including promoter-associated factors, chromatin regulatory proteins, and telomeric factors (reviewed by [19]). Nuclear PI enzymes and metabolites play roles in the control of VSG expression in coordination with ES regulatory molecules [2,4]. The regulation entails local control by PIP5Pase of nuclear levels of PIP3 and PIP3 binding by repressor activator protein 1 (RAP1) [4]. PIP5Pase and RAP1 interact within a 0.9 MDa protein complex and associate with telomeric ESs and are enriched in 70 bp and telomeric repeats [4]. The derepression of silent VSG genes resulting from knockdown of either RAP1 or PIP5Pase underscores their functional association [2,21]. Moreover, PIP5Pase activity, i.e., dephosphorylation of PIP3 5-position phosphate, is required for regulation as shown by the derepression of all silent VSG genes upon mutation of PIP5Pase to loss of catalytic function [4]. Furthermore, PIP3, but not other phosphoinositides, binds to RAP1, and mutations that catalytically inactivate PIP5Pase also alter RAP1 association with ESs resulting in RAP1 dissociation from telomeric repeats [2,4]. The changes in RAP1 and PIP5Pase association with ES chromatin likely affect chromatin organization and result in transcription of silent VSG genes. Hence, the PIP5Pase enzyme controls the silencing of all but 1 telomeric VSG ES, and the regulatory mechanism involves PIP5Pase catalysis, which controls the RAP1 silencing function via PIP3 levels. PIP3 association with RAP1 affects RAP1 interactions with chromatin and thus chromatin organization and its accessibility to RNAP I transcription elongation. Therefore, the PIP3 levels available to PIP5Pase, and locally to RAP1, appear to be a key element of the regulatory mechanism. Other cellular regulatory processes may entail similar mechanisms of control by PI enzymes and targets, namely, via control of subcellular metabolite fluxes that result from substrate interconversions, binding, and transport.

The control of VSG ES transcription in *T*. *brucei* is also affected by PIP5K and PLC, which are located at the plasma membrane [2]. Knockdown of PIP5K, which synthesizes PIP2, and overexpression of PLC, which cleaves PIP2 into diacylglycerol and IP3, both result in the loss of VSG allelic exclusion [2]. Furthermore, the temporary knockdown of PIP5K results in the switching of VSGs by transcriptional and recombination mechanisms. How the regulation of phosphoinositides at the plasma membrane affects nuclear ES regulatory processes involved in VSG expression and switching remains unknown. However, this functional association between the 2 plasma membrane enzymes and VSG ES transcription and switching in the nucleus implies that transcriptional control of VSG ESs is linked to a signal transduction system, which may also entail environmental sensing. Hence, the PI system activation might result in a signal transduction cascade that controls the nuclear machinery involved in VSG transcription and recombination. The signal transduction might be like the conserved IP3 signaling in eukaryotes since *T*. *brucei* expresses all components of the IP3 signaling pathway [34,37] (Table 1). Still, it may also involve other regulatory proteins such as kinases and phosphatases. The interactome of PIP5Pase and RAP1 identified many nuclear kinases and phosphatases that could play a role in this process [4]. Hence, the PI system regulates nuclear processes in a fashion that coordinates ES transcription with other cellular functions.

The PI metabolites function in the control of nuclear processes is not limited to trypanosomes [2,4] but is also evident in yeast [40,64] and mammals [42,45]. The mechanisms by which PIs control nuclear processes are still emerging. However, the existing data indicate that they involve specific PI binding by proteins, which controls protein activity or interactions with other proteins, DNA, or RNA [45,53,65]. The result is the regulation of various nuclear processes including transcription factor activation [42,66], RNAP I transcription [43,51], RNA polyadenylation [65], RNA transport [44,64], and chromatin modifications [45]. It remains unknown whether PI metabolites are transported to the nucleus after synthesis in endoplasmic reticulum (ER)/Golgi or whether they are synthesized in the nucleus [67]. Nevertheless, the nuclear localization of PI enzymes indicates that PI levels are regulated in the nucleus of various organisms [2,40,66,67]. The role of PIs in the regulation of nuclear processes in trypanosomes, yeast, and mammalian cells indicates that PI regulatory functions in the nucleus originated early in eukaryotic evolution and likely diversified to regulate specialized processes in these organisms.

## IP kinases and phosphatases role in *T*. *brucei* life stage development

Numerous regulatory processes control the various cell-wide changes that occur between *T*. *brucei* developmental stages [3,15,17,68–70]. Importantly, however, these processes must be functionally integrated within the cells overall regulatory systems. These include processes that regulate energy generating systems [3,15], cell surface composition [2,71,72], and cell division [22,73,74]. The PI system functions in the regulation of BF to PF development [3]. Knockdown or mutation that inactivates IPMK, the enzyme that phosphorylates inositol positions 3 and 6 of IP3 and generates IP4 and D-myo-inositol 1,3,4,5,6-pentakisphosphate (IP5) [3,34], results in the development of dividing slender to nondividing stumpy BFs and then PFs. This transition is accompanied by decreased VSG expression and transient PAD1 expression, which is followed by procyclin expression, a switch from glycolysis to oxphos, and morphological changes (Fig 1) [3]. On the other hand, knockdown of inositol polyphosphate 5-phosphatase (IP5Pase) is not lethal but results in morphological changes that resemble stumpies and in a decrease in pyruvate release without effects on ATP production [3]. This is unlike knockdown of IPMK, or its mutation to catalytic inactivity, which results in BF development to PFs that have a functional, i.e., inhibitor sensitive, oxphos system [3]. These enzymes, both of which are cytoplasmic, may function at a developmental juncture. The IP5Pase knockdown may tilt the regulatory network toward cell division–arrested stumpy BFs that are responsive to the mammalian environment. Responsiveness to the mammalian environment is also implied by the cell surface location of PIP5K and PLC [2]. Notably, PLC produces IP3, which is the IPMK substrate. The IPMK knockdown may tilt the regulatory network toward cells that are responsive to the PF growth environment and mitochondrial development including generation of a fully functional oxphos system that requires regulation of RNA editing (discussed below). Such responsiveness is also implied by citrate-cis aconitate (CCA) enhancement of BF to PF development in a dose-dependent manner after IPMK knockdown [3]. Hence, the PI system regulation of development is integrated within the cells systems that sense and transduce environmental conditions, which includes adenosine monophosphate (AMP) and cyclic AMP (cAMP) signaling [22,68,75], CCA [3,25], and its response to cell density [22,23].

The PI system must function in coordination with other cellular signaling and regulatory processes involved in the control of *T*. *brucei* development. cAMP and AMP are involved in the regulation of *T*. *brucei* development from slender to stumpy BFs. They function by activation of AMP-activated kinase (AMPK), which stimulates the development of slender to stumpy BFs [22,68]. Moreover, AMP and cAMP or their analogs inhibit the target of rapamycin 4 (TOR4) complex, which negatively regulates the development of slender to stumpy BFs [75]. On the other hand, regulation of stumpy BFs to PFs involves protein tyrosine phosphatase 1 (PTP1) and the glycosomal Ser/Thr phosphatase PTP1-interacting protein of 39 kDa (PIP39), both of which are involved in the differentiation of BFs to PFs via CCA stimuli [76,77]. These proteins are part of the regulatory network involved in the control of *T*. *brucei* development, and they might function in concert with IP5Pase and IPMK to control cellular responses to environmental changes, e.g., changes associated with the transition from host to vector and/or changes that are inherent to the cell developmental program.

## IPMK control of metabolic switch between developmental stages

The PI system controls processes that occur in a coordinated fashion during cell development [3,78]. Such regulatory processes are evident in the life stage developmental changes in *T*. *brucei*, which entail shifting between different primary means of energy generation as they alternate between glycolysis in BFs in the mammalian host and amino acids consumption and oxphos in PFs in the insect vector (discussed in Introduction) [3,31,79]. The precise mechanisms that control the glycosomal and cytoplasmic composition of glycolytic enzymes and the differential editing are unknown, but they certainly are affected by processes that involve regulation by the PI system [3]. For example, mutations that inactivate *T*. *brucei* IPMK catalysis result in cells that switch cellular energy metabolism from glycolysis to oxphos in BFs. Importantly, these changes occur in BFs and precede their development into insect stage PFs [3]. IPMK knockdown or catalytic inactivation results in an approximately 4-fold increase in intracellular ATP levels [3]. These changes correlate with an increase in the expression of respiratory chain genes, including those encoding proteins of complex II, III, and IV, which are expressed in PFs but not in BFs. Moreover, the increased ATP levels are sensitive to inhibitors of oxphos proteins that are typically expressed in PFs but not in BFs. Notably, the production of a functional oxphos system in BFs requires differential editing of mitochondrial mRNAs. Hence, significant changes in the expression of genes from both the nuclear and mitochondrial genomes occur upon perturbation of IPMK, and consequently, functional changes in the energy generation system [3]. A potential mechanism for IP regulation of metabolic changes could involve IPs binding to RNA-binding proteins (RBPs), and hence affect mRNA stability or translational control. Our data indicate that perturbation of the IP regulatory network affects stage-specific gene expression and changes in expression of several RBPs, including RBP6, RBP7, and RBP10 [3]. These RBPs control the expression of hundreds of genes including those involved in energy generation systems such as glycolysis and oxphos, the coordination of surface protein expression (e.g., procyclins), and morphological changes [3,69,70,80,81]. An alternative mechanism involves the direct interaction of IPs with metabolic and signaling proteins to control protein function and thus regulate cell metabolism. In *T*. *brucei*, IP3 and IP4 bind to over 100 proteins from BFs [3]. Proteins bound by IP3 are generally associated with cell signaling and motility, while proteins bound by IP4 are associated with cell metabolism and protein synthesis and degradation, which implies that soluble IPs have multiple cellular regulatory roles in *T*. *brucei* [3]. The potential mechanisms are not mutually exclusive, and *T*. *brucei* could employ various strategies to regulate energy metabolism and other developmental processes. Other soluble IPs such as D-myo-inositol 1,2,3,4,5,6-hexakisphosphate (IP6), D-myo-inositol 5-diphospho 1,2,3,4,6-pentakisphosphate (IP7), and D-myo-Inositol 1,5-bis(diphosphate) 2,3,4,6-tetrakisphosphate (IP8), which are produced in yeast [82], also have regulatory functions, although they are not yet completely studied in trypanosomes [83].

Control of the switch between glycolysis and oxphos by the PI system appears to be conserved in eukaryotes, albeit differentially adapted by evolution between organisms [3,61]. The knockdown of IPMK in yeast also results in a switch from oxphos to glycolysis [61]. In addition, the Warburg effect in mammalian cancer cells, in which the cells employ oxidative glycolysis, resembles the consequence of IPMK perturbation [61]. The molecular mechanisms underlying these regulatory processes in trypanosomes, yeast, and mammals are unknown. The involvement of control of transcription occurs in yeast because the activity of the GCR1 glycolytic transcription complex is affected by the knockdown of IPMK and IP6K [40,58,61]. In addition, the yeast chromatin–remodeling SWItch/Sucrose Non-Fermentable (SWI/SNF) complex is regulated by IP4 [61]. Also, IP5 and IP6 inhibit nucleosome mobilization by the chromatin remodeling complexes nucleosome remodeling factor, ISWI (Imitation Switch) chromatin-remodeling complex adenosine triphosphatase (ATPase) ISW2, and chromatin-remodeling ATPase INO80 complexes [40]. These results imply that changes in IP metabolite levels and perhaps fluxes affect gene transcription in these cells. However, trypanosomes do not control transcription initiation and generally control gene expression posttranscriptionally, e.g., RNA turnover or translational control [84,85]. In *T*. *brucei*, RBPs may act at the RNA level by controlling mRNA stability, degradation, or translation and result in a similar phenotypic outcome as control of transcription initiation in yeast. However, the IP regulation may also act posttranscriptionally even in organisms that employ control of transcription initiation [44,52,64]. Hence, IPs appear to have conserved roles in the switch between glycolysis and oxphos, but different or additional regulatory mechanisms seem to be employed in different organisms.

## PI kinases: Regulation of organelle biogenesis and trafficking

Phosphoinositides play a key role in organelle biogenesis and vesicle trafficking in eukaryotes, including endosome to lysosome and endosome to Golgi trafficking [86–88]. In *T*. *brucei*, regulation of protein trafficking is essential for surface expression and turnover of proteins such as VSGs and procyclins [89]. In *T*. *brucei*, the phosphatidylinositol synthase (PIS) and CDP-diacylglycerol synthetase (CDS) enzymes are located in the ER and Golgi [35,90] (Table 1 and Fig 3) where they synthesize PIs that are distributed to other cellular compartments, e.g., plasma membrane and organelles. Subsequently, PIs are phosphorylated by PI kinases generating a variety of PI phosphates (PIPs) that are distributed to other cellular compartments by mechanisms that are yet poorly understood [91]. In BFs, PIP, PIP2, and PIP3 metabolites are on the plasma membrane and elsewhere [2], e.g., in endosomal compartments with PIP2 [i.e., PI(4,5)P2] at the flagellar pocket [7] and PI(3,5)P2 in lysosomes [5]. The knockdown of a phosphatidylinositol 3-kinase (PI3K) homolog of yeast vacuolar protein sorting 34p (vps34p) in BFs impairs receptor-mediated endocytosis of transferrin and concanavalin A to lysosomes and the export of VSGs to the cell surface [6]. Interestingly, the high rate of VSG endocytosis from the surface to lysosomes is implicated in the removal and degradation of surface-bound immunocomplexes (e.g., antibodies and complement factors), which aids parasite immune evasion [92]. The PI3K knockdown parasites also fail to segregate the Golgi during mitosis despite correct replication and segregation of kinetoplasts and basal bodies [6]. Furthermore, the knockdown of the phosphatidylinositol 4-kinase beta (PI4Kβ) in BFs results in Golgi abnormalities and the mislocalization of lysosomal and flagellar pocket proteins [93]. It also results in the accumulation of intracellular vesicles, which suggests defects in vesicle trafficking [93]. In BFs, the knockdown of PIP5K results in decreased levels of plasma membrane PIP2 and affects the parasite shape, i.e., cells become round [2] and impairs endocytosis [7]. On the other hand, the knockdown of phosphatidylinositol 3-phosphate 5-kinase (PI3P5K) in BFs, the ortholog of yeast lysosomal Fab1, decreases the levels of PI(3,5)P2 metabolites and results in defects in the late endosomal degradation pathway [5]. Hence, the PI system plays a role in the regulation of vesicle trafficking, organelle biogenesis, and recycling of surface proteins. Although these regulatory functions are somewhat conserved with other eukaryotes, their role in the correct delivery of VSG proteins to the surface and the high rate of VSG surface recycling are intertwined with antigenic variation and the removal of surface-bound immunocomplexes, which helps the parasite to evade the host immune response. Hence, the PI system has roles in vesicle trafficking and organelle biogenesis and likely evolved specialized regulatory features in *T*. *brucei* that contribute to host immune evasion.

## Evolutionary significance and functional diversity of the PI system in eukaryotes

The PI enzymes are conserved among eukaryotes but expanded in metazoans, where they likely play similar regulatory roles but are adapted to the various differentiated cell types (Fig 2C). The diversity of the PI system regulatory functions might reflect differences in the expression of genes encoding PI enzymes, differences in enzyme activities, or posttranslational regulation of enzyme function. In addition, the set of genes encoding enzymes of the PI system in each organism’s genome may contribute to its diversity of functions. Analysis of genes encoding PI enzymes in the genome of multiple organisms such as archaea, bacteria, protozoa, yeast, and metazoans such as plants, worms, and humans shows dramatic differences in the overall number of genes between these organisms and the class of enzymes that each organism genome encodes (Fig 2C). For example, archaea and bacteria such as *Escherichia coli* and *Mycobacteria tuberculosis* contain a small subset of genes encoding enzymes involved in the synthesis of inositol and phosphoinositides. Moreover, they lack most enzymes involved in the phosphorylation or dephosphorylation of inositol, as well as genes encoding proteins involved in IP3 signaling such as PLC and IP3R. The presence of genes involved in PI synthesis in prokaryotes and archaea might be related to the expression of surface glycoconjugates that contain inositol [94,95]. Since archaea and bacteria apparently lack most of the conserved PI kinases and phosphatases, it is likely that the regulatory functions that PI metabolites have in eukaryotes are absent in prokaryotes and archaea. In contrast, protozoan eukaryotes such as *Giardia Lamblia*, *T*. *brucei*, or *Plasmodium falciparum* have a substantial set of PI enzymes. This large set of enzymes correlates with numerous regulatory functions that involve the PI system in protozoa, e.g., regulation of VSG gene expression and life stage development in *T*. *brucei* [2,3], *T*. *cruzi* differentiation and host cell interaction [54], and development in *Plasmodium* sp. [55]. Interestingly, no IP3Rs have been identified in Apicomplexan parasites, possibly due to their having divergent IP3Rs [59], although there is evidence for IP3 signaling in *Plasmodium* [55,56]. A cyclic guanosine monophosphate (cGMP)-dependent protein kinase G controls IP3 intracellular production through regulation of lipid kinases and function in malaria parasite motility and development [55]. The IP3 function in the control of Ca^2+^ levels seems also conserved between protozoa and metazoans [50], although in *T*. *brucei*, the IP3R localizes in acidocalcisomes rather than in the ER, as it occurs in metazoans. In addition to IP3, other soluble IPs may also have a conserved regulatory functions in protozoans and metazoans [3,61]. The early evolutionary divergence of trypanosomes from other eukaryotes suggests that roles for PIs in the regulation of cells’ metabolic balance, homeostasis, environmental sensing, and development might have originated early in evolution. Metazoans, including humans, contain many genes for PI enzymes with regulatory and signaling functions, including those encoding PLC, PI kinases, and phosphatases [50,52,60,65,78,96]. The expansion in the number of PI enzyme paralogs from unicellular protozoa to metazoans reflects the more complex regulatory systems in multicellular organisms. This implies additional processes that are regulated by the PI system, such as those that control cell-specific PI enzyme subcellular compartmentalization, differential gene expression, splicing, regulation of posttranslational modifications, and feedback controls [97]. This expanded gene number and additional levels of regulation also reflect tissue-specific gene expression, multiple cell types, and the complex development that these organisms undergo. Hence, differences in the number of PI system genes among various organisms suggest that the PI system evolved to accommodate the complexity of regulatory functions in eukaryotes and may echo the diversity of functional roles in which PI enzymes are involved.

## Concluding remarks

All cells function as integrated units that maintain homeostasis while responding to environmental changes and periodically undergoing numerous coordinated changes during development and differentiation. The processes that control homeostasis and differentiation function individually within each cell in populations of unicellular organisms, albeit these cells may interact with, as well as respond to, their environment. The multicellular metazoan organisms, including humans, are much more complex but nevertheless function as a unit and employ the PI system within their regulatory processes. Their greater complexity includes the added dimensions of specific interactions of variously differentiated cells, as amply illustrated by the immune system, and their organization into tissues and organs. This greater complexity in metazoans is reflected in the expansion of the PI system. Nevertheless, many of the regulatory principles are likely to be conserved between *T*. *brucei* and metazoans, including humans.

The PI system in *T*. *brucei*, and likely in many other organisms, has also evolved to regulate specialized processes. This is evinced by the role of the PI system in the regulation of VSG expression and switching in *T*. *brucei*, and it is conceivable that part of this signaling and regulatory mechanism is conserved among other eukaryotes, especially those that uses antigenic variation for host immune evasion. The PI system has also evolved to function integrated within the specific biology and developmental features of each organism. The role of IPMK in energy generating systems is illustrative: it regulates energy metabolism in many eukaryotes, but while in yeast and mammals it involves transcription control of metabolic genes, in *T*. *brucei*, it seems to involve posttranscriptional regulatory processes. The presence of PI system kinases and phosphatases allows for a regulatory system that is tunable and responsive to multiple cellular conditions, as it seems to occur with the regulation of *T*. *brucei* life stage development. The ability of these metabolites to functionally associate with proteins, RNAs, and lipids, and hence be distributed to many subcellular compartments, may be a critical characteristic that give them such a widespread regulatory function in eukaryotes.

The PI regulatory system provides attractive drug targets in pathogens [34,98], especially where they differ from homologs in the host, and the functions in the host are less vital than in the pathogen. Besides, inhibition of key PI enzymes in the pathogens may disrupt essential processes or those that allow the pathogen to escape host defenses, e.g., antigenic variation [2,4]. Findings in *T*. *brucei* may provide insights for drug development, including in other eukaryotes.

## References

[1] Pires-daSilva A, Sommer RJ. The evolution of signalling pathways in animal development. Nat Rev Genet. 2003;4(1):39–49. Epub 2003 Jan 2. 10.1038/nrg977 . 12509752

[2] Cestari I, Stuart K. Inositol phosphate pathway controls transcription of telomeric expression sites in trypanosomes. Proc Natl Acad Sci U S A. 2015;112(21):E2803–E2812. 10.1073/pnas.1501206112 25964327 PMC4450425

[3] Cestari I, Anupama A, Stuart K. Inositol polyphosphate multikinase regulation of Trypanosoma brucei life stage development. Mol Biol Cell. 2018;29(9):1137–1152. Epub 2018 Mar 9. 10.1091/mbc.E17-08-0515 29514930 PMC5921579

[4] Cestari I, McLeland-Wieser H, Stuart K. Nuclear phosphatidylinositol 5-phosphatase is essential for allelic exclusion of variant surface glycoprotein genes in trypanosomes. Mol Cell Biol. 2019;39(3). Epub 2018 Nov 14. 10.1128/MCB.00395-18 . 30420356 PMC6336139

[5] Gilden JK, Umaer K, Kruzel EK, Hecht O, Correa RO, Mansfield JM, et al The role of the PI(3,5)P2 kinase TbFab1 in endo/lysosomal trafficking in Trypanosoma brucei. Mol Biochem Parasitol. 2017;214:52–61. Epub 2017 Mar 31. 10.1016/j.molbiopara.2017.03.005 28356223 PMC5474170

[6] Hall BS, Gabernet-Castello C, Voak A, Goulding D, Natesan SK, Field MC. TbVps34, the trypanosome orthologue of Vps34, is required for Golgi complex segregation. J Biol Chem. 2006;281(37):27600–27612. 10.1074/jbc.M602183200 . 16835237

[7] Demmel L, Schmidt K, Lucast L, Havlicek K, Zankel A, Koestler T, et al The endocytic activity of the flagellar pocket in Trypanosoma brucei is regulated by an adjacent phosphatidylinositol phosphate kinase. J Cell Sci. 2014;127(Pt 10):2351–2364. 10.1242/jcs.146894 24639465 PMC4021478

[8] Capewell P, Cren-Travaille C, Marchesi F, Johnston P, Clucas C, Benson RA, et al The skin is a significant but overlooked anatomical reservoir for vector-borne African trypanosomes. Elife. 2016;5 Epub 2016 Sep 23. 10.7554/eLife.17716 27653219 PMC5065312

[9] Trindade S, Rijo-Ferreira F, Carvalho T, Pinto-Neves D, Guegan F, Aresta-Branco F, et al Trypanosoma brucei parasites occupy and functionally adapt to the adipose tissue in mice. Cell Host Microbe. 2016;19(6):837–848. Epub 2016 May 31. 10.1016/j.chom.2016.05.002 27237364 PMC4906371

[10] Dovey HF, Parsons M, Wang CC. Biogenesis of glycosomes of Trypanosoma brucei: an in vitro model of 3-phosphoglycerate kinase import. Proc Natl Acad Sci U S A. 1988;85(8):2598–2602. 10.1073/pnas.85.8.2598 3282231 PMC280045

[11] Blattner J, Helfert S, Michels P, Clayton C. Compartmentation of phosphoglycerate kinase in Trypanosoma brucei plays a critical role in parasite energy metabolism. Proc Natl Acad Sci U S A. 1998;95(20):11596–11600. 10.1073/pnas.95.20.11596 9751711 PMC21686

[12] Schnaufer A, Clark-Walker GD, Steinberg AG, Stuart K. The F1-ATP synthase complex in bloodstream stage trypanosomes has an unusual and essential function. EMBO J. 2005;24(23):4029–4040. 10.1038/sj.emboj.7600862 16270030 PMC1356303

[13] Stuart KD, Schnaufer A, Ernst NL, Panigrahi AK. Complex management: RNA editing in trypanosomes. Trends Biochem Sci. 2005;30(2):97–105. 10.1016/j.tibs.2004.12.006 . 15691655

[14] Souza AE, Myler PJ, Stuart K. Maxicircle CR1 transcripts of Trypanosoma brucei are edited and developmentally regulated and encode a putative iron-sulfur protein homologous to an NADH dehydrogenase subunit. Mol Cell Biol. 1992;12(5):2100–2107. Epub 1992 May 1. 10.1128/mcb.12.5.2100 1373807 PMC364381

[15] Feagin JE, Stuart K. Developmental aspects of uridine addition within mitochondrial transcripts of Trypanosoma brucei. Mol Cell Biol. 1988;8(3):1259–1265. 10.1128/mcb.8.3.1259 2452974 PMC363271

[16] Feagin JE, Abraham JM, Stuart K. Extensive editing of the cytochrome c oxidase III transcript in Trypanosoma brucei. Cell. 1988;53(3):413–422. 10.1016/0092-8674(88)90161-4 . 2452697

[17] Bienen EJ, Saric M, Pollakis G, Grady RW, Clarkson AB Jr. Mitochondrial development in Trypanosoma brucei brucei transitional bloodstream forms. Mol Biochem Parasitol. 1991;45(2):185–192. 10.1016/0166-6851(91)90085-k . 1645458

[18] Abraham JM, Feagin JE, Stuart K. Characterization of cytochrome c oxidase III transcripts that are edited only in the 3' region. Cell. 1988;55(2):267–272. Epub 1988 Oct 21. 10.1016/0092-8674(88)90049-9 . 2844415

[19] Cestari I, Stuart K. Transcriptional regulation of telomeric expression sites and antigenic variation in Trypanosomes. Curr Genomics. 2018;19(2):119–132. Epub 2018 Mar 2. 10.2174/1389202918666170911161831 29491740 PMC5814960

[20] Horn D, Cross GA. A developmentally regulated position effect at a telomeric locus in Trypanosoma brucei. Cell. 1995;83(4):555–561. 10.1016/0092-8674(95)90095-0 . 7585958

[21] Yang X, Figueiredo LM, Espinal A, Okubo E, Li B. RAP1 is essential for silencing telomeric variant surface glycoprotein genes in Trypanosoma brucei. Cell. 2009;137(1):99–109. 10.1016/j.cell.2009.01.037 19345190 PMC2673096

[22] Vassella E, Reuner B, Yutzy B, Boshart M. Differentiation of African trypanosomes is controlled by a density sensing mechanism which signals cell cycle arrest via the cAMP pathway. J Cell Sci. 1997;110(Pt 21):2661–2671. Epub 1998 Jan 14. . 9427384 10.1242/jcs.110.21.2661

[23] Rojas F, Silvester E, Young J, Milne R, Tettey M, Houston DR, et al Oligopeptide signaling through TbGPR89 drives Trypanosome quorum sensing. Cell. 2018 Epub 2018 Dec 7. 10.1016/j.cell.2018.10.041 . 30503212 PMC6333907

[24] MacGregor P, Savill NJ, Hall D, Matthews KR. Transmission stages dominate trypanosome within-host dynamics during chronic infections. Cell Host Microbe. 2011;9(4):310–318. 10.1016/j.chom.2011.03.013 21501830 PMC3094754

[25] Dean S, Marchetti R, Kirk K, Matthews KR. A surface transporter family conveys the trypanosome differentiation signal. Nature. 2009;459(7244):213–217. 10.1038/nature07997 19444208 PMC2685892

[26] Reuner B, Vassella E, Yutzy B, Boshart M. Cell density triggers slender to stumpy differentiation of Trypanosoma brucei bloodstream forms in culture. Mol Biochem Parasitol. 1997;90(1):269–280. Epub 1998 Mar 13. 10.1016/s0166-6851(97)00160-6 . 9497048

[27] Capewell P, Monk S, Ivens A, Macgregor P, Fenn K, Walrad P, et al Regulation of Trypanosoma brucei total and polysomal mRNA during development within its mammalian host. PLoS ONE. 2013;8(6):e67069 10.1371/journal.pone.0067069 23840587 PMC3694164

[28] Feagin JE, Jasmer DP, Stuart K. Differential mitochondrial gene expression between slender and stumpy bloodforms of Trypanosoma brucei. Mol Biochem Parasitol. 1986;20(3):207–214. Epub 1986 Sep 1. 10.1016/0166-6851(86)90100-3 . 2429179

[29] Bienen EJ, Webster P, Fish WR. Trypanosoma (Nannomonas) congolense: changes in respiratory metabolism during the life cycle. Exp Parasitol. 1991;73(4):403–412. Epub 1991 Nov 1. 10.1016/0014-4894(91)90064-4 . 1720394

[30] Jensen BC, Ramasamy G, Vasconcelos EJ, Ingolia NT, Myler PJ, Parsons M. Extensive stage-regulation of translation revealed by ribosome profiling of Trypanosoma brucei. BMC Genomics. 2014;15:911 10.1186/1471-2164-15-911 25331479 PMC4210626

[31] Ginger ML. Niche metabolism in parasitic protozoa. Philos Trans R Soc Lond Ser B Biol Sci. 2006;361(1465):101–118. 10.1098/rstb.2005.1756 16553311 PMC1626543

[32] Koslowsky DJ, Riley GR, Feagin JE, Stuart K. Guide RNAs for transcripts with developmentally regulated RNA editing are present in both life cycle stages of Trypanosoma brucei. Mol Cell Biol. 1992;12(5):2043–2049. Epub 1992 May 1. 10.1128/mcb.12.5.2043 1373804 PMC364375

[33] Rotureau B, Van Den Abbeele J. Through the dark continent: African trypanosome development in the tsetse fly. Front Cell Infect Microbiol. 2013;3:53 Epub 2013 Sep 26. 10.3389/fcimb.2013.00053 24066283 PMC3776139

[34] Cestari I, Haas P, Moretti NS, Schenkman S, Stuart K. Chemogenetic characterization of inositol phosphate metabolic pathway reveals druggable enzymes for targeting kinetoplastid parasites. Cell Chem Biol. 2016;23(5):608–617. 10.1016/j.chembiol.2016.03.015 27133314 PMC4876820

[35] Martin KL, Smith TK. Phosphatidylinositol synthesis is essential in bloodstream form Trypanosoma brucei. Biochem J. 2006;396(2):287–295. 10.1042/BJ20051825 16475982 PMC1462709

[36] King-Keller S, Moore CA, Docampo R, Moreno SN. Ca2+ regulation of Trypanosoma brucei phosphoinositide phospholipase C. Eukaryot Cell. 2015;14(5):486–94. Epub 2015 Mar 15. 10.1128/EC.00019-15 25769297 PMC4421009

[37] Huang G, Bartlett PJ, Thomas AP, Moreno SN, Docampo R. Acidocalcisomes of Trypanosoma brucei have an inositol 1,4,5-trisphosphate receptor that is required for growth and infectivity. Proc Natl Acad Sci U S A. 2013;110(5):1887–1892. 10.1073/pnas.1216955110 23319604 PMC3562765

[38] Hammond GR, Balla T. Polyphosphoinositide binding domains: Key to inositol lipid biology. Biochim Biophys Acta. 2015;1851(6):746–758. Epub 2015 Mar 4. 10.1016/j.bbalip.2015.02.013 25732852 PMC4380703

[39] Wu M, Chong LS, Perlman DH, Resnick AC, Fiedler D. Inositol polyphosphates intersect with signaling and metabolic networks via two distinct mechanisms. Proc Natl Acad Sci U S A. 2016;113(44):E6757–E6765. Epub 2016 Nov 3. 10.1073/pnas.1606853113 27791083 PMC5098652

[40] Steger DJ, Haswell ES, Miller AL, Wente SR, O'Shea EK. Regulation of chromatin remodeling by inositol polyphosphates. Science. 2003;299(5603):114–116. 10.1126/science.1078062 12434012 PMC1458531

[41] Chakraborty A, Kim S, Snyder SH. Inositol pyrophosphates as mammalian cell signals. Sci Signal. 2011;4(188):re1 10.1126/scisignal.2001958 21878680 PMC3667551

[42] Blind RD, Sablin EP, Kuchenbecker KM, Chiu HJ, Deacon AM, Das D, et al The signaling phospholipid PIP3 creates a new interaction surface on the nuclear receptor SF-1. Proc Natl Acad Sci U S A. 2014;111(42):15054–15059. Epub 2014 Oct 8. 10.1073/pnas.1416740111 25288771 PMC4210282

[43] Sobol M, Yildirim S, Philimonenko VV, Marasek P, Castano E, Hozak P. UBF complexes with phosphatidylinositol 4,5-bisphosphate in nucleolar organizer regions regardless of ongoing RNA polymerase I activity. Nucleus. 2013;4(6):478–486. 10.4161/nucl.27154 24513678 PMC3925692

[44] Adams RL, Mason AC, Glass L, Aditi, Wente SR. Nup42 and IP6 coordinate Gle1 stimulation of Dbp5/DDX19B for mRNA export in yeast and human cells. Traffic. 2017;18(12):776–790. Epub 2017 Sep 5. 10.1111/tra.12526 28869701 PMC5677552

[45] Watson PJ, Fairall L, Santos GM, Schwabe JW. Structure of HDAC3 bound to co-repressor and inositol tetraphosphate. Nature. 2012;481(7381):335–340. 10.1038/nature10728 22230954 PMC3272448

[46] Strahl T, Hama H, DeWald DB, Thorner J. Yeast phosphatidylinositol 4-kinase, Pik1, has essential roles at the Golgi and in the nucleus. J Cell Biol. 2005;171(6):967–979. Epub 2005 Dec 21. 10.1083/jcb.200504104 16365163 PMC1382337

[47] Hammond GR, Hong Y. Phosphoinositides and membrane targeting in cell polarity. Cold Spring Harb Perspect Biol. 2018;10(2). Epub 2017 Mar 8. 10.1101/cshperspect.a027938 . 28264819 PMC5793754

[48] Lystad AH, Simonsen A. Phosphoinositide-binding proteins in autophagy. FEBS Lett. 2016;590(15):2454–2468. Epub 2016 Jul 9. 10.1002/1873-3468.12286 . 27391591

[49] Hawse WF, Cattley RT. T cells transduce T-cell receptor signal strength by generating different phosphatidylinositols. J Biol Chem. 2019;294(13):4793–4805. Epub 2019 Jan 30. 10.1074/jbc.RA118.006524 30692200 PMC6442064

[50] Mikoshiba K. Role of IP3 receptor signaling in cell functions and diseases. Adv Biol Regul. 2015;57:217–227. Epub 2014 Dec 17. 10.1016/j.jbior.2014.10.001 . 25497594

[51] Yildirim S, Castano E, Sobol M, Philimonenko VV, Dzijak R, Venit T, et al Involvement of phosphatidylinositol 4,5-bisphosphate in RNA polymerase I transcription. J Cell Sci. 2013;126(Pt 12):2730–2739. 10.1242/jcs.123661 . 23591814

[52] Wickramasinghe VO, Savill JM, Chavali S, Jonsdottir AB, Rajendra E, Gruner T, et al Human inositol polyphosphate multikinase regulates transcript-selective nuclear mRNA export to preserve genome integrity. Mol Cell. 2013;51(6):737–750. Epub 2013 Oct 1. 10.1016/j.molcel.2013.08.031 . 24074953

[53] Sablin EP, Blind RD, Uthayaruban R, Chiu HJ, Deacon AM, Das D, et al Structure of liver receptor homolog-1 (NR5A2) with PIP3 hormone bound in the ligand binding pocket. J Struct Biol. 2015;192(3):342–348. Epub 2015 Sep 30. 10.1016/j.jsb.2015.09.012 26416531 PMC4651778

[54] Hashimoto M, Enomoto M, Morales J, Kurebayashi N, Sakurai T, Hashimoto T, et al Inositol 1,4,5-trisphosphate receptor regulates replication, differentiation, infectivity and virulence of the parasitic protist Trypanosoma cruzi. Mol Microbiol. 2013;87(6):1133–1150. 10.1111/mmi.12155 . 23320762

[55] Brochet M, Collins MO, Smith TK, Thompson E, Sebastian S, Volkmann K, et al Phosphoinositide metabolism links cGMP-dependent protein kinase G to essential Ca(2)(+) signals at key decision points in the life cycle of malaria parasites. PLoS Biol. 2014;12(3):e1001806 Epub 2014 Mar 7. 10.1371/journal.pbio.1001806 24594931 PMC3942320

[56] Alves E, Bartlett PJ, Garcia CR, Thomas AP. Melatonin and IP3-induced Ca2+ release from intracellular stores in the malaria parasite Plasmodium falciparum within infected red blood cells. J Biol Chem. 2011;286(7):5905–5912. Epub 2010 Dec 15. 10.1074/jbc.M110.188474 21149448 PMC3037703

[57] Jungmichel S, Sylvestersen KB, Choudhary C, Nguyen S, Mann M, Nielsen ML. Specificity and commonality of the phosphoinositide-binding proteome analyzed by quantitative mass spectrometry. Cell Rep. 2014;6(3):578–591. 10.1016/j.celrep.2013.12.038 . 24462288

[58] Odom AR, Stahlberg A, Wente SR, York JD. A role for nuclear inositol 1,4,5-trisphosphate kinase in transcriptional control. Science. 2000;287(5460):2026–2029. Epub 2000 Mar 17. 10.1126/science.287.5460.2026 . 10720331

[59] Garcia CRS, Alves E, Pereira PHS, Bartlett PJ, Thomas AP, Mikoshiba K, et al InsP3 signaling in apicomplexan parasites. Curr Top Med Chem. 2017;17(19):2158–2165. Epub 2017 Feb 1. 10.2174/1568026617666170130121042 28137231 PMC5490149

[60] Blind RD. Disentangling biological signaling networks by dynamic coupling of signaling lipids to modifying enzymes. Adv Biol Regul. 2014;54:25–38. Epub 2013 Nov 2. 10.1016/j.jbior.2013.09.015 24176936 PMC3946453

[61] Szijgyarto Z, Garedew A, Azevedo C, Saiardi A. Influence of inositol pyrophosphates on cellular energy dynamics. Science. 2011;334(6057):802–805. 10.1126/science.1211908 . 22076377

[62] Herman PK, Emr SD. Characterization of VPS34, a gene required for vacuolar protein sorting and vacuole segregation in Saccharomyces cerevisiae. Mol Cell Biol. 1990;10(12):6742–54. Epub 1990 Dec 1. 10.1128/mcb.10.12.6742 2247081 PMC362952

[63] Clayton C. The regulation of trypanosome gene expression by RNA-binding proteins. PLoS Pathog. 2013;9(11):e1003680 10.1371/journal.ppat.1003680 24244152 PMC3820711

[64] York JD, Odom AR, Murphy R, Ives EB, Wente SR. A phospholipase C-dependent inositol polyphosphate kinase pathway required for efficient messenger RNA export. Science. 1999;285(5424):96–100. Epub 1999 Jul 3. 10.1126/science.285.5424.96 . 10390371

[65] Mellman DL, Gonzales ML, Song C, Barlow CA, Wang P, Kendziorski C, et al A PtdIns4,5P2-regulated nuclear poly(A) polymerase controls expression of select mRNAs. Nature. 2008;451(7181):1013–1017. 10.1038/nature06666 . 18288197

[66] Blind RD, Suzawa M, Ingraham HA. Direct modification and activation of a nuclear receptor-PIP(2) complex by the inositol lipid kinase IPMK. Sci Signal. 2012;5(229):ra44 10.1126/scisignal.2003111 22715467 PMC3395721

[67] Irvine RF. Nuclear lipid signalling. Nat Rev Mol Cell Biol. 2003;4(5):349–360. 10.1038/nrm1100 . 12728269

[68] Saldivia M, Ceballos-Perez G, Bart JM, Navarro M. The AMPKalpha1 pathway positively regulates the developmental transition from proliferation to quiescence in Trypanosoma brucei. Cell Rep. 2016;17(3):660–670. 10.1016/j.celrep.2016.09.041 27732844 PMC5074416

[69] Kolev NG, Ramey-Butler K, Cross GA, Ullu E, Tschudi C. Developmental progression to infectivity in Trypanosoma brucei triggered by an RNA-binding protein. Science. 2012;338(6112):1352–1353. 10.1126/science.1229641 23224556 PMC3664091

[70] Mony BM, MacGregor P, Ivens A, Rojas F, Cowton A, Young J, et al Genome-wide dissection of the quorum sensing signalling pathway in Trypanosoma brucei. Nature. 2014;505(7485):681–685. 10.1038/nature12864 24336212 PMC3908871

[71] Kramer S, Queiroz R, Ellis L, Hoheisel JD, Clayton C, Carrington M. The RNA helicase DHH1 is central to the correct expression of many developmentally regulated mRNAs in trypanosomes. J Cell Sci. 2010;123(Pt 5):699–711. Epub 2010 Feb 4. 10.1242/jcs.058511 20124414 PMC2823576

[72] Glover L, Hutchinson S, Alsford S, Horn D. VEX1 controls the allelic exclusion required for antigenic variation in trypanosomes. Proc Natl Acad Sci U S A. 2016;113(26):7225–7230. 10.1073/pnas.1600344113 27226299 PMC4932947

[73] Matthews KR, Gull K. Commitment to differentiation and cell cycle re-entry are coincident but separable events in the transformation of African trypanosomes from their bloodstream to their insect form. J Cell Sci. 1997;110(Pt 20):2609–2618. Epub 1998 Feb 12. . 9372450 10.1242/jcs.110.20.2609

[74] Gale M Jr, Carter V, Parsons M. Translational control mediates the developmental regulation of the Trypanosoma brucei Nrk protein kinase. J Biol Chem. 1994;269(50):31659–31665. . 7989338

[75] Barquilla A, Saldivia M, Diaz R, Bart JM, Vidal I, Calvo E, et al Third target of rapamycin complex negatively regulates development of quiescence in Trypanosoma brucei. Proc Natl Acad Sci U S A. 2012;109(36):14399–14404. 10.1073/pnas.1210465109 22908264 PMC3437835

[76] Szoor B, Ruberto I, Burchmore R, Matthews KR. A novel phosphatase cascade regulates differentiation in Trypanosoma brucei via a glycosomal signaling pathway. Genes Dev. 2010;24(12):1306–1316. 10.1101/gad.570310 20551176 PMC2885665

[77] Szoor B, Dyer NA, Ruberto I, Acosta-Serrano A, Matthews KR. Independent pathways can transduce the life-cycle differentiation signal in Trypanosoma brucei. PLoS Pathog. 2013;9(10):e1003689 10.1371/journal.ppat.1003689 24146622 PMC3798605

[78] Seeds AM, Tsui MM, Sunu C, Spana EP, York JD. Inositol phosphate kinase 2 is required for imaginal disc development in Drosophila. Proc Natl Acad Sci U S A. 2015;112(51):15660–15665. Epub 2015 Dec 10. 10.1073/pnas.1514684112 26647185 PMC4697392

[79] Mantilla BS, Marchese L, Casas-Sanchez A, Dyer NA, Ejeh N, Biran M, et al Proline metabolism is essential for Trypanosoma brucei brucei survival in the tsetse vector. PLoS Pathog. 2017;13(1):e1006158 10.1371/journal.ppat.1006158 . 28114403 PMC5289646

[80] Wurst M, Seliger B, Jha BA, Klein C, Queiroz R, Clayton C. Expression of the RNA recognition motif protein RBP10 promotes a bloodstream-form transcript pattern in Trypanosoma brucei. Mol Microbiol. 2012;83(5):1048–1063. 10.1111/j.1365-2958.2012.07988.x . 22296558

[81] Jha BA, Gazestani VH, Yip CW, Salavati R. The DRBD13 RNA binding protein is involved in the insect-stage differentiation process of Trypanosoma brucei. FEBS Lett. 2015;589(15):1966–1974. Epub 2015 Jun 2. 10.1016/j.febslet.2015.05.036 . 26028502

[82] Mulugu S, Bai W, Fridy PC, Bastidas RJ, Otto JC, Dollins DE, et al A conserved family of enzymes that phosphorylate inositol hexakisphosphate. Science. 2007;316(5821):106–109. 10.1126/science.1139099 . 17412958

[83] Cordeiro CD, Saiardi A, Docampo R. The inositol pyrophosphate synthesis pathway in Trypanosoma brucei is linked to polyphosphate synthesis in acidocalcisomes. Mol Microbiol. 2017;106(2):319–333. Epub 2017 Aug 10. 10.1111/mmi.13766 28792096 PMC5630508

[84] De Gaudenzi JG, Noe G, Campo VA, Frasch AC, Cassola A. Gene expression regulation in trypanosomatids. Essays Biochem. 2011;51:31–46. Epub 2011 Oct 26. 10.1042/bse0510031 . 22023440

[85] Gazestani VH, Lu Z, Salavati R. Deciphering RNA regulatory elements in trypanosomatids: one piece at a time or genome-wide? Trends Parasitol. 2014;30(5):234–240. Epub 2014/03/20. 10.1016/j.pt.2014.02.008 . 24642036

[86] Nascimbeni AC, Giordano F, Dupont N, Grasso D, Vaccaro MI, Codogno P, et al ER-plasma membrane contact sites contribute to autophagosome biogenesis by regulation of local PI3P synthesis. EMBO J. 2017;36(14):2018–2033. Epub 2017 May 28. 10.15252/embj.201797006 28550152 PMC5509996

[87] Dong R, Saheki Y, Swarup S, Lucast L, Harper JW, De Camilli P. Endosome-ER contacts control actin nucleation and retromer function through VAP-dependent regulation of PI4P. Cell. 2016;166(2):408–423. Epub 2016 Jul 16. 10.1016/j.cell.2016.06.037 27419871 PMC4963242

[88] Billcliff PG, Noakes CJ, Mehta ZB, Yan G, Mak L, Woscholski R, et al OCRL1 engages with the F-BAR protein pacsin 2 to promote biogenesis of membrane-trafficking intermediates. Mol Biol Cell. 2016;27(1):90–107. Epub 2015 Oct 30. 10.1091/mbc.E15-06-0329 26510499 PMC4694765

[89] Venkatesh D, Zhang N, Zoltner M, Del Pino RC, Field MC. Evolution of protein trafficking in kinetoplastid parasites: complexity and pathogenesis. Traffic. 2018;19(11):803–812. Epub 2018 Jul 6. 10.1111/tra.12601 . 29974581

[90] Lilley AC, Major L, Young S, Stark MJ, Smith TK. The essential roles of cytidine diphosphate-diacylglycerol synthase in bloodstream form Trypanosoma brucei. Mol Microbiol. 2014;92(3):453–470. 10.1111/mmi.12553 24533860 PMC4114554

[91] Cockcroft S. Phosphatidylinositol transfer proteins couple lipid transport to phosphoinositide synthesis. Semin Cell Dev Biol. 2001;12(2):183–191. Epub 2001 Apr 9. 10.1006/scdb.2000.0235 . 11292384

[92] Engstler M, Pfohl T, Herminghaus S, Boshart M, Wiegertjes G, Heddergott N, et al Hydrodynamic flow-mediated protein sorting on the cell surface of trypanosomes. Cell. 2007;131(3):505–515. Epub 2007 Nov 6. 10.1016/j.cell.2007.08.046 . 17981118

[93] Rodgers MJ, Albanesi JP, Phillips MA. Phosphatidylinositol 4-kinase III-beta is required for Golgi maintenance and cytokinesis in Trypanosoma brucei. Eukaryot Cell. 2007;6(7):1108–1118. 10.1128/EC.00107-07 17483288 PMC1951100

[94] Morii H, Ogawa M, Fukuda K, Taniguchi H. Ubiquitous distribution of phosphatidylinositol phosphate synthase and archaetidylinositol phosphate synthase in Bacteria and Archaea, which contain inositol phospholipid. Biochem Biophys Res Commun. 2014;443(1):86–90. Epub 2013 Nov 26. 10.1016/j.bbrc.2013.11.054 . 24269814

[95] Salman M, Lonsdale JT, Besra GS, Brennan PJ. Phosphatidylinositol synthesis in mycobacteria. Biochim Biophys Acta. 1999;1436(3):437–450. Epub 1999 Feb 16. 10.1016/s0005-2760(98)00151-9 . 9989274

[96] Bang S, Kim S, Dailey MJ, Chen Y, Moran TH, Snyder SH, et al AMP-activated protein kinase is physiologically regulated by inositol polyphosphate multikinase. Proc Natl Acad Sci U S A. 2012;109(2):616–620. 10.1073/pnas.1119751109 22203993 PMC3258619

[97] Andersen JF, Ribeiro JM. A secreted salivary inositol polyphosphate 5-phosphatase from a blood-feeding insect: allosteric activation by soluble phosphoinositides and phosphatidylserine. Biochemistry. 2006;45(17):5450–5457. Epub 2006 Apr 26. 10.1021/bi052444j . 16634626

[98] McNamara CW, Lee MC, Lim CS, Lim SH, Roland J, Simon O, et al Targeting Plasmodium PI(4)K to eliminate malaria. Nature. 2013;504(7479):248–253. Epub 2013 Nov 29. 10.1038/nature12782 24284631 PMC3940870

[99] Gonzalez-Salgado A, Steinmann ME, Greganova E, Rauch M, Maser P, Sigel E, et al myo-Inositol uptake is essential for bulk inositol phospholipid but not glycosylphosphatidylinositol synthesis in Trypanosoma brucei. J Biol Chem. 2012;287(16):13313–13323. 10.1074/jbc.M112.344812 22351763 PMC3340000

[100] Russo-Abrahao T, Koeller CM, Steinmann ME, Silva-Rito S, Marins-Lucena T, Alves-Bezerra M, et al H(+)-dependent inorganic phosphate uptake in Trypanosoma brucei is influenced by myo-inositol transporter. J Bioenerg Biomembr. 2017;49(2):183–194. Epub 2017 Feb 12. 10.1007/s10863-017-9695-y . 28185085

[101] Martin KL, Smith TK. The myo-inositol-1-phosphate synthase gene is essential in Trypanosoma brucei. Biochem Soc Trans. 2005;33(Pt 5):983–985. 10.1042/BST20050983 . 16246027

[102] Barquilla A, Crespo JL, Navarro M. Rapamycin inhibits trypanosome cell growth by preventing TOR complex 2 formation. Proc Natl Acad Sci U S A. 2008;105(38):14579–14584. Epub 2008 Sep 18. 10.1073/pnas.0802668105 18796613 PMC2567229

[103] de Jesus TC, Tonelli RR, Nardelli SC, da Silva AL, Motta MC, Girard-Dias W, et al Target of rapamycin (TOR)-like 1 kinase is involved in the control of polyphosphate levels and acidocalcisome maintenance in Trypanosoma brucei. J Biol Chem. 2010;285(31):24131–24140. Epub 2010 May 25. 10.1074/jbc.M110.120212 20495004 PMC2911323

[104] Jones NG, Thomas EB, Brown E, Dickens NJ, Hammarton TC, Mottram JC. Regulators of Trypanosoma brucei cell cycle progression and differentiation identified using a kinome-wide RNAi screen. PLoS Pathog. 2014;10(1):e1003886 10.1371/journal.ppat.1003886 24453978 PMC3894213

